# Grass-legume mixture and nitrogen application improve yield, quality, and water and nitrogen utilization efficiency of grazed pastures in the loess plateau

**DOI:** 10.3389/fpls.2023.1088849

**Published:** 2023-01-31

**Authors:** Ranran Xu, Wei Shi, Muhammad Kamran, Shenghua Chang, Qianmin Jia, Fujiang Hou

**Affiliations:** ^1^ State Key Laboratory of Herbage Improvement and Grassland Agro-ecosystems; Key Laboratory of Grassland Livestock Industry Innovation, Ministry of Agriculture and Rural Affairs; Engineering Research Center of Grassland Industry, Ministry of Education; College of Pastoral Agricultural Science and Technology, Lanzhou University, Lanzhou, China; ^2^ Tropical Crops Genetic Resources Institute, Chinese Academy of Tropical Agricultural Sciences, Haikou, China; ^3^ Hainan Key Laboratory for Sustainable Utilization of Tropical Bioresources, College of Tropical Crops, Hainan University, Haikou, China

**Keywords:** grazing, water and nitrogen utilization efficiency, alfalfa, forage yield, quality

## Abstract

Grazing on cultivated grassland is a green agricultural model. However, in China's Loess Plateau, the type of cultivated grassland suitable for grazing and the amount of nitrogen application is still unclear, which has led to the failure of this model to be widely implemented. In this context, we set up an experiment using three grass planting types, including monoculture of alfalfa (Medicago sativa L.), monoculture of brome (Bromus inermis L.), and mixed planting of the two forages. Under each planting type, there were six management measures: grazing and no nitrogen application (GN1), grazing and 80 kg ha^-1^ nitrogen application (GN2), grazing and 160 kg ha^-1^ nitrogen application (GN3), cutting and no nitrogen application (MN1), cutting and 80 kg ha^-1^ nitrogen application (MN2), and cutting and 160 kg ha^-1^ nitrogen application (MN3). To explore the impacts of these treatments on pastures, we studied the effects on the yield, quality, and water use efficiency of the three cultivated grasslands. Results showed that alfalfa monoculture and alfalfa-brome mixed sowing grassland resulted in significantly higher hay yield, crude protein yield, water use efficiency (WUE), precipitation use efficiency (PUE), nitrogen use efficiency (NUE), and agronomic efficiency of nitrogen (AEN) as compared to brome monoculture grassland. In addition, the crude protein, ether extract, and crude ash content of alfalfa monoculture and alfalfa-brome mixture were increased significantly while the contents of neutral detergent fiber (NDF) were reduced, thereby increasing the relative feed value (RFV) during the two years. The forage hay yield, crude protein yield, ether extract, crude ash content, RFV, PUE, and WUE were significantly higher with GN1, GN2, and GN3 treatments than that with MN1 treatment. In contrast, the NDF and acid detergent fiber (ADF) content was significantly lower than the MN1 treatment. Furthermore, the fresh forage yield, crude protein yield, PUE, and WUE of GN3 treatment were significantly higher than that of GN1 and GN2 treatments in both years, while the NUE and AEN were significantly higher in GN2 and GN3 treatments than that of MN3 treatment. Based on these results, alfalfa-brome mixed cropping with the application of 160 kg ha^-1^ nitrogen under grazing conditions is an appropriate management practice for improving the forage yield, quality, and water- and nitrogen utilization efficiency of cultivated grassland in the Loess Plateau of China. This integrated management model is applicable to the cultivation and utilization of mixed grassland on nutrient-poor land in the Loess Plateau.

## 1 Introduction

Soil erosion has resulted in a gradual increase in land barrenness and deterioration of the ecological environment, severely restricting the development of local industrial and agricultural production (Zhou et al., 2013). Planting perennial pastures in the loess plateau region is of practical importance because it will not only relieve the grazing pressure of natural grasslands but will also solve the problem of insufficient forage for livestock in the winter and spring seasons (Hou et al., 2008). In addition, cultivating grasslands in the Loess Plateau is an effective way of changing the land use patterns in the region and promoting ecological and economic development (Komarek et al., 2015). Compared to monoculture grasses, alfalfa and gramineous mixed grassland can not only increase the forage yield (Sanderson et al., 2005; Deak et al., 2009) but will also improve the nutritional quality of pasture (Tekeli and Ates, 2005). In addition, grazing on the grass-legume mixed grassland can improve grassland productivity by better-storing moisture and inorganic salt in the soil (Pykälä, 2005). In alfalfa-brome mixed sowing grassland, brome can utilize the nitrogen fixed by alfalfa, improve the N_2_ fixation efficiency of alfalfa, and promote the absorption of nitrogen by brome (Lahiri et al., 2009; Li et al., 2011).

In addition, fertilization is one of the important agronomic measure for improving the yield and quality of pastures. Nitrogen application in cultivated grassland can improve the yield and crude protein content of dry matter, thus improving the nutritional quality of pasture (Mbatha and Ward, 2010; Tomić et al., 2012). Nitrogen is a major component of protein synthesis, and increased application of nitrogen fertilizer has been known to have positive effects on the nutrient absorption and utilization of cultivated grassland (Xiong et al., 2013; Gou et al., 2016). However, nitrogen application has a threshold effect in regulating crop growth, i.e. excessive nitrogen application is not conducive to improving crop growth and yield (Mon et al., 2016; Wang et al., 2017; Wang H. et al., 2018). The crude protein content and yield of Urochloa brizantha and Marandu pastures were increased with the increase in nitrogen application rates during each grazing cycle, while the content of neutral detergent fiber (NDF) was reduced, and in the case of continuous grazing, moderate nitrogen application (180 kg^-1^ N) resulted in high yield and quality forage (Campos et al., 2016; Delevatti et al., 2019). Brueck et al. (2010) showed that nitrogen application can improve the water use efficiency (WUE) of pasture regardless of water supply (Mckenzie et al., 2006; Brueck et al., 2010). However, Li et al. (2003) suggested that measuring soil nitrogen supply capacity and plant nitrogen demand at different stages, and providing timely and appropriate nitrogen fertilizer supply can improve the soil nutrient status, production performance, and sustainable productivity of cultivated grassland.

Moderate grazing is an effective way of managing grassland vegetation (Huntsinger et al., 2007). Moderate grazing is not only a management measure for preventing habitat loss or fragmentation but also a way for improving grassland biodiversity (Bartolome et al., 2014). Previous studies have found an increase in the biomass and crude protein content of the above-ground plants with the increase in the stocking rate (Schönbach et al., 2012a; Müller et al., 2014; Ren et al., 2016), while the content of neutral detergent fiber only decreased in the short term with the increase of stocking rate (Schönbach et al., 2009). Schönbach et al., 2012a showed that compared with light and heavy grazing, the available feed biomass under moderate grazing increased by 2 to 3 times, and plant nutrients were improved. However, some studies have reported that grazing has little impact on forage nutritional quality, but forages in grazing land had higher crude protein content in the late rainy season (Mbatha and Ward, 2010). Grazing not only increases the forage yield and improves nutritive quality but also the water use efficiency (Fenetahun et al., 2020). Peng et al. (2007) reported that the water use efficiency (WUE) of Cleistogenes squarrosa, Agropyron cristatum, and Potentilla acaulis increased significantly as the grazing intensity increased, reaching the highest value at moderate grazing intensity. Because of trampling disturbs soil by enhancing evaporation of water, sheep’s dunk or urine might contribute to increase soil moisture (Peng et al., 2007). Under grazing conditions, *Leymus chinensis* is more sensitive to water deficit. It responds to grazing disturbance by reducing transpiration rate and improving WUE (D’Andrea et al., 2017). Grazing improves the water use efficiency of pasture, possibly due to the concentration of grazing grass roots to the surface soil, thus increasing the absorption and utilization of water by plant roots (Zheng et al., 2011).

Intensive grazing not only increase stocking rates and reduce costs but also improve soil moisture status and reduce soil erosion (Sone et al., 2020). Several studies have shown that grazing in the Loess Plateau, where “returning cropland to grassland” is practiced, significantly reduced soil erosion and improved soil characteristics (Wang et al., 2009; Chen et al., 2015; Yang and Lu, 2018). The results of Yu et al. (2019) also showed that light grazing not only reduced soil erosion but also protected natural resources. Without disrupting the soil environment, grazing management may benefit the economic development of local animal husbandry and increase the income of local farmers to ensure food security and resolve conflicts of interest between agricultural development and nature protection (Sparovek et al., 2010; Spera, 2017). In the Loess Plateau of China, reasonable grazing of cultivated grassland is a sustainable grassland management model, and one of the strategies to achieve the dual goals of ecological and economic benefits (Wang and Zhang, 2003). However, most studies on grazing activities are concentrated on natural grasslands. There are few studies on grazing management of cultivated grasslands in the Loess Plateau, and the appropriate amount of nitrogen fertilizer for grass-legume mixed grassland is still uncertain. Therefore, this study explored the effect of grazing combined with nitrogen application on the yield, quality, water, and nitrogen utilization of cultivated grassland under the grassland types of monoculture alfalfa, monoculture brome, and mixed planting of the two forages. The objectives were to (a) explore the advantages of grass-legume mixed grassland compared with pasture monoculture; (b) clarify the appropriate management measures for improving the yield, quality, WUE, and NUE of cultivated grassland in the Loess Plateau.

## 2 Materials and methods

### 2.1 Overview of the research area

The study area is located in Huan xian Grassland Agricultural Experiment Station of Lanzhou University, Qingyang City, Gansu Province (36° 17′10″ N, 107° 31′36 ″E), with an altitude of 1218 m. It is a hilly and gully area on the Loess Plateau in eastern Gansu, with a typical river valley agricultural production system and semi-arid continental climate. The average annual rainfall is 430 mm and is mostly concentrated in July-September, accounting for 58.2% of the total annual precipitation. The annual potential evaporation reaches 1850 mm; the annual mean temperature is 9.2°C. The frost-free period is 165 days and the annual mean sunshine duration is 2596.2 hours. Compared with the 30-year average rainfall, 2019 (505.5 mm) was considered as a wet year, while 2020 (434.1 mm) as a normal year. Before the commencement of the experiment, the soil (0-20 cm soil layer) had the following soil properties; pH value of 8.5, soil organic carbon of 4.9 g kg^-1^, the total nitrogen content of 0.67 g kg^-1^, the available phosphorus content of 11.6 mg kg^-1^, available potassium content 142 mg kg^-1^.

### 2.2 Experimental design and field management

This study was arranged in a split-plot design. The main plot factor includes three grassland types: monoculture of alfalfa, monoculture of brome, and mixed cropping of alfalfa and brome. The sub-plot factor included six management measures: grazing and 0 kg ha^-1^ nitrogen application level of (GN1), grazing and 80 kg ha^-1^ nitrogen application level of (GN2), grazing and 160 kg ha^-1^ nitrogen application level of (GN3), cutting and 0 kg ha^-1^ nitrogen application level (MN1), cutting and 80 kg ha^-1^ nitrogen application level (MN2), and cutting and 160 kg ha^-1^ nitrogen application level (MN3). In this way the experiment consisted of a total of 18 treatments with three replications, resulting in a total of 54 treatment plots. The area of each treatment plot was 40 m^2^ (5 m × 8 m), separated by a 1 m wide isolation belt. In addition, fences were set up around the grazing plots. The grassland was planted in August 2017, and 225 kg ha^-1^ diammonium hydrogen phosphate was used as the base fertilizer for each sowing plot. No irrigation was provided during the experimental period. The alfalfa variety Qianjing (*Medicago sativa* L. ‘Vison’), and the brome American variety (*Bromus inermis* L. ‘Vns’) were used in the experiment. Before planting, ploughing (30 cm depth) was employed for removing weeds. Seeds were sown by drill sowing method at a row spacing of 30 cm, and planting depth of 2 to 3 cm. The seeding rate for monoculture alfalfa was 30 kg ha^-1^, for monoculture brome was 45 kg ha^-1^, while that of alfalfa and brome mixed grassland was 15.0 and 22.5 kg ha^-1^, respectively. In both years, no fertilizers were applied to the N1 treated plots. The N2 plots were fertilized on 3rd June 2019 and 8th June 2020; while the N3 plots were fertilized on 3rd June and 1st August 2019, and 8th June and 5th August 2020. Urea (CH_4_N_2_O) was used as a nitrogen source and 80 kg N ha^-1^ was applied in trenches each time. Rotational grazing was carried out approximately every 30 days. There were 81 sheep in total and 9 sheep were allotted to each plot, and all the forages in the plot were grazed. Overall, 12 grazing cycles were performed during the two years. The cutting treatment was carried out at the flowering stage of the leguminous family or the heading period of the gramineous family. The stubble height was maintained at 5 cm, and 6 cuttings were carried out in two years, corresponding to May 21, July 23, September 24 in 2019, and May 24, July 15, and September 6 in 2020.

### 2.3 Sampling and measurements

#### 2.3.1 Determination of forage yield and nutritional quality

For grazing treatments, samplings were carried out before each grazing, and for cutting treatments, samplings were performed at the leguminous flowering period or gramineous heading period. For cutting, 1 m^2^ area was randomly selected at three different locations in each plot and immediately weighed for the fresh weight. The samples were put in a mesh bag, transported to the lab, and later oven-dried at 65°C for 48 h or until constant weight, and dry matter yield was determined. The seasonal total fresh grass and hay yields were the sum of the fresh and hay yields of each cutting throughout the growing period. The crude protein yield was calculated using the following formula:


$$Y_{P}\left(\text{t}\;{\text{ha}}^{‐1}\right)\;=\;C_{P}\times Y\;\times \;0.01$$

where Y_P_ is crude protein yield (t·ha^-1^), C_P_ is crude protein content (%), Y is hay yield (kg ha^-1^).

The dried samples were crushed and were analyzed for the determination of nutrient values. The content of ether extract (ether extract, EE) was determined by the Soxhlet ether extraction method using an ether extract analyzer (XT15, Ankom, America) (Association of Official Analytical Chemists, 2000). Crude protein (CP) content was determined by the Kjeldahl method using the nitrogen analyzer (Kjeltec 2300, Foss Tecator, Sweden) (Jee, 1995). The crude ash content was determined by the incineration method in a muffle furnace (LE14/16/R6, Nabertherm, Germany) at 550°C (Van Soest, 1994). The content of neutral detergent fiber (NDF) and acid detergent fiber (ADF) was measured in a fiber analyzer (2000, Ankom, America) using the paradigm detergent fiber analysis method (Van Soest et al., 1991). The relative feeding value (RFV) was calculated using the following formulae (Kamran et al., 2022; Kamran et al., 2023):


RFV = (120/NDF) × (88.9−0.799ADF)/1.29

Whereas NDF and ADF represent the neutral and acid detergent fiber, respectively.

#### 2.3.2 Water utilization status

To determine the soil quality content of the soil samples were collected at recovering and withering date of the pasture A soil drill was used for collecting soil samples from 0-200 cm soil layer, each with a 20 cm increment. The soil was placed aluminum box and oven-dried at 105°C for 24 h or to a constant weight. The soil moisture contents in terms of soil water storage (SWS) were calculated following the formula (Wu et al., 2015):


$$\text{SWS}\left(\text{mm}\right)=\sum_{1}^{n}h_{i}\times p_{i}\times b_{i}$$

Where SWS is the soil water storage capacity (mm), hi is the depth of the soil layer (cm); *p_i_* is the soil bulk density of the soil layer (g cm^-3^); *b_i_* is the absolute soil of the soil layer Mass moisture content (%); n is the number of soil layers.

The water consumption in terms of evapotranspiration (ET) from the field was calculated using the formula (Huang et al., 2005) is as follows:


ET (mm) = P + I + C +W1 − W2 – D – R

Where P (mm) is the rainfall during the growth period, I (mm) is the irrigation volume, C (mm) is the amount of groundwater at the roots zones, and W1 (mm) is the water storage at recovering date of 0-200 cm soil layer, W2 (mm) is the water storage at the withering date of 0-200 cm soil layer, D (mm) is the water discharge outside the roots, and R (mm) is the surface runoff loss. However, the runoff loss can be ignored as the test site is relatively flat, and ridges around the plot prevent runoff. The groundwater level of the test site was deeper than 80 m. As no irrigation was provided, therefore, the amount of groundwater flowing into the roots, rainfall-runoff loss, irrigation, and water discharge beyond the roots can be ignored (Zhao et al., 2014).

The calculation formulas for precipitation use efficiency and water use efficiency (are as follows Huang et al., 2005):


$${\text{PUE (kg ha}}^{-1}{\text{ mm}}^{-1}\text{) = Y/P}$$

$${\text{WUE (kg ha}}^{-1}{\text{ mm}}^{-1}\text{) = Y/ET}$$

Whereas PUE is the precipitation use efficiency (kg ha^-1^ mm^-1^), WUE is water use efficiency (kg ha^-1^ mm^-1^), Y is hay yield (kg ha^-1^), and ET is field water consumption (mm), P is the rainfall during the growth period (mm).

#### 2.3.3 Nitrogen utilization status

Determination of forage nitrogen content was carried out using FOSS-NIRS DS 2500 (Denmark) Near-Infrared Spectrometer. The nitrogen uptake, nitrogen use efficiency, and agronomic efficiency of nitrogen were calculated using the following formulas (Guo et al., 2014):


$${\text{N}}_{\text{U}}\;(\text{kg}\;{\text{ha}}^{-1})={\text{N}}_{\text{C}}*\text{Y}$$

$$NUE\;\left(kg\;{kg}^{-1}\right)\;=\;\left(U_{N}–U_{0}\right)/F_{N}$$

$$AEN\;\left(kg\;{kg}^{-1}\right)\;=\;\left(Y_{N}–Y_{0}\right)/F_{N}$$

where, N_U_ and Nc are nitrogen absorption (kg ha^-1^) and nitrogen content (%); NUE is nitrogen utilization efficiency (kg kg^-1^), U_N_ and U_0_ are nitrogen absorption (kg ha^−1^) in the nitrogen application treatment and no nitrogen application treatment; AEN is nitrogen agronomic efficiency (kg kg^-1^), Y_N_ and Y_0_ are yield (kg ha^−1^) of nitrogen application treatment and no nitrogen application treatment; and F_N_ is the N rate (kg ha^−1^) in N application treatments.

### 2.4 Data processing and statistical methods

**Figure 1:**
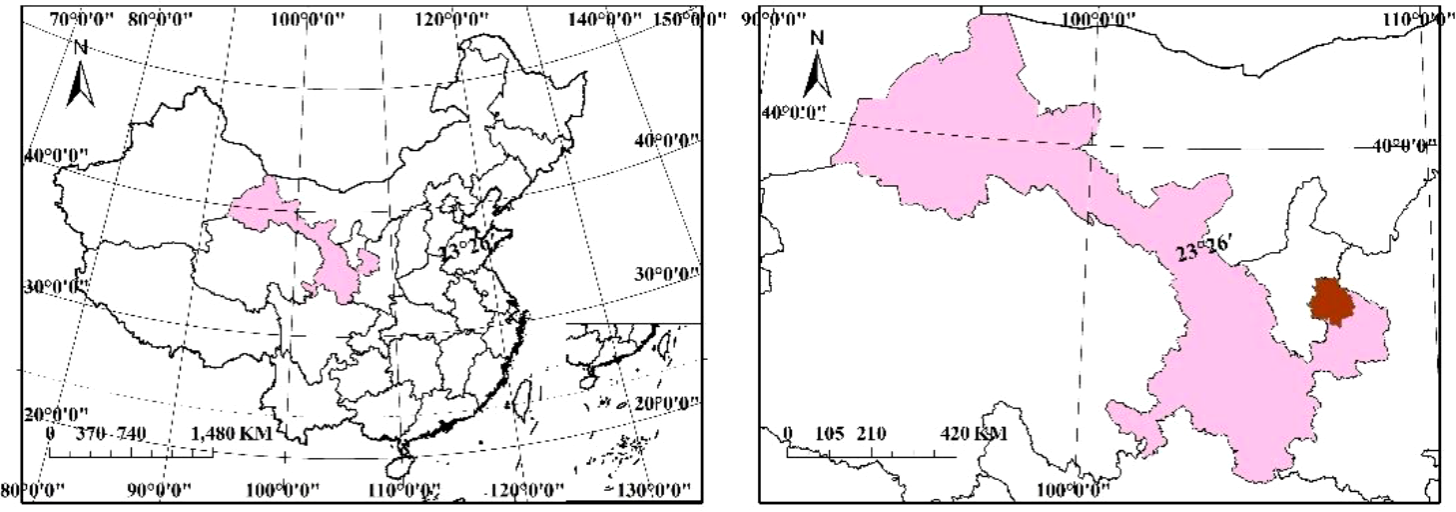
The geographical location of Huanxian test station.

**Figure 2:**
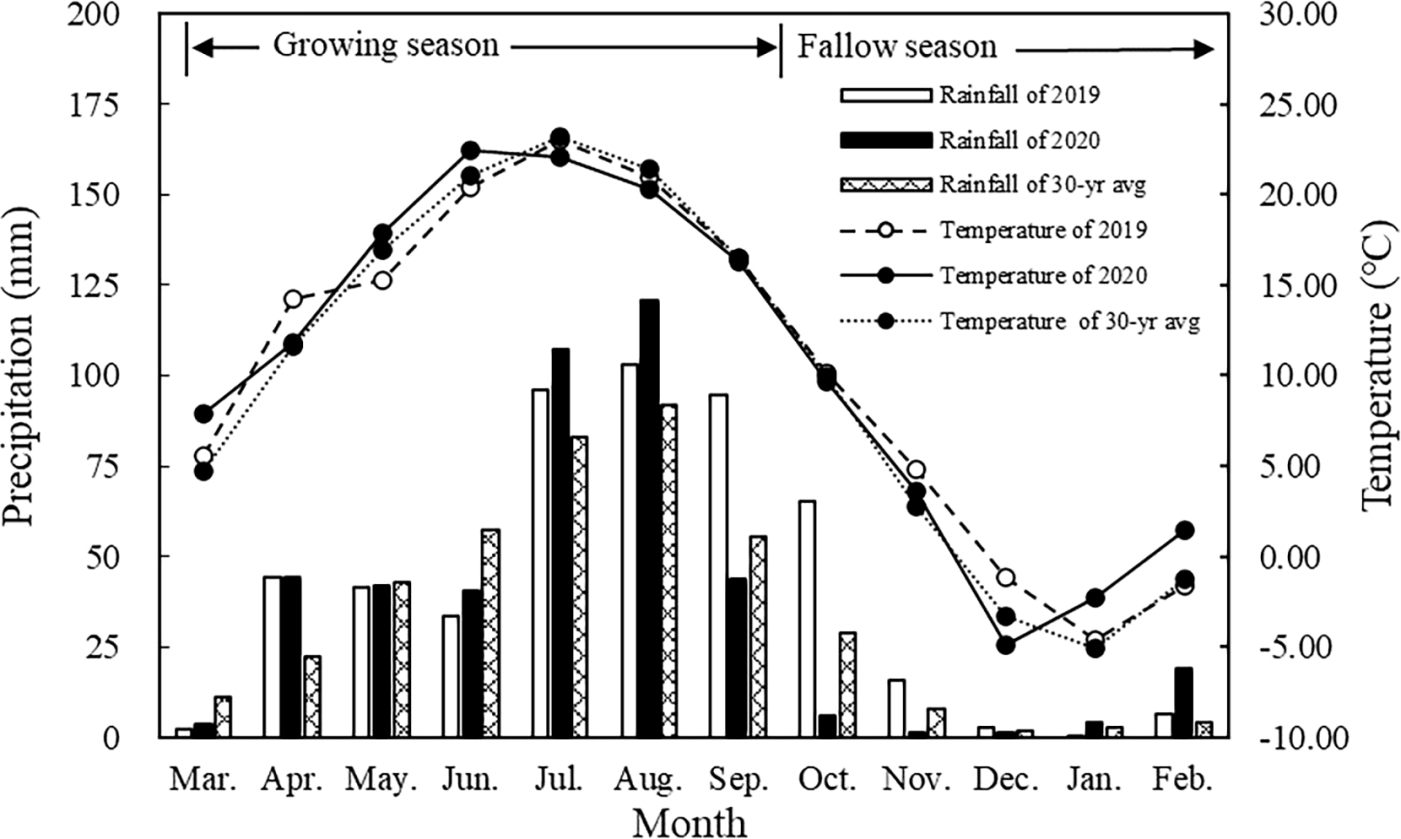
Monthly average precipitation and temperature in Huanxian experimental station.

Microsoft Excel 2010 was used to process the data and draw figures. After testing the data normality and homogeneity of variance, the data follows normal distribution and meets the homogeneity test of variance, the statistical software SPSS 24 (IBM, Chicago, IL, USA) was employed for the analysis of variance. To analyze the effects of different treatments on fresh hay yield, nutrient content, crude protein yield, relative feed value, water use efficiency, precipitation use efficiency, nitrogen use efficiency, and agronomic efficiency of nitrogen of forage, the Tukey significant difference test was employed for multiple comparisons (*P* ≤ 0.05). **Figures 1**, **2**.

## 3 Results

### 3.1 Fresh hay yield

The ANOVA results indicated significant effects (*P* ≤ 0.01) of the year (Y), grassland type (GT), and management style (MM) on fresh biomass of pastures (**Table 1**). The interaction effects were also significant (*P* ≤ 0.01). The fresh biomass yield of all treatments in 2019 was significantly higher by 20.5% than that in 2020. Among the grassland types, alfalfa-brome mixed sowing resulted in the highest fresh biomass compared to their monocultures (**Table S1**). In addition, the GN3 showed the most significant effect among the various management styles (**Table S1**).

**Table 1:** ANOVA results for the effect of the year, grassland type, management style, and their interactions on fresh forage biomass, hay yield, crude protein yield, the content of crude protein, crude fat, crude ash, acid detergent fiber (ADF), neutral detergent fiber (NDF), and relative feed value (RFV); soil water storage, evapotranspiration, water use efficiency (WUE), precipitation use efficiency (PUE); nitrogen uptake, nitrogen content, nitrogen use efficiency (NUE) and agronomic efficiency of nitrogen (AEN) in 2019-2020.

Parameters	Year (Y)	Grassland Type (GT)	Management Style (MM)	Y×GT	Y×MM	GT×MM	Y×GT×MM
Fresh forage biomass	335.63^**^	1545.99^**^	383.22^**^	211.68^**^	9.39^**^	12.98^**^	4.43^**^
Hay yield	36.79^**^	1221.14^**^	347.26^**^	35.61^**^	6.42^**^	9.96^**^	4.38^**^
Crude protein yield	20.25^**^	2131.82^**^	441.93^**^	18.55^**^	7.06^**^	25.20^**^	4.64^**^
Crude protein	3.99^*^	438.56^**^	17.67^**^	0.72^ns^	1.14^ns^	1.44^ns^	0.16^ns^
Crude fat	842.18^**^	410.89^**^	32.01^**^	36.81^**^	3.12^*^	0.31^ns^	0.20^ns^
Crude ash	174.03^**^	777.14^**^	11.42^**^	64.73^**^	3.07^*^	1.09^ns^	0.85^ns^
ADF	31.29^**^	11.38^**^	33.22^**^	6.59^**^	3.26^*^	0.46^ns^	0.43^ns^
NDF	25.51^**^	243.17^**^	15.56^**^	54.68^**^	0.90^ns^	0.41^ns^	0.34^ns^
RFV	81.74^**^	292.27^**^	40.81^**^	75.51^**^	3.45^**^	0.74^ns^	0.76^ns^
Soil water storage at recovering	0.76^ns^	103.99^**^	17.96^**^	55.35^**^	7.57^**^	2.12^*^	2.06^*^
Soil water storage at withering	4.97^*^	188.75^**^	24.40^**^	21.77^**^	13.45^**^	2.46^*^	2.62^**^
Evapotranspiration	292.60^**^	0.85^ns^	0.15^ns^	10.03^**^	1.23^ns^	0.57^ns^	0.69^ns^
WUE	120.74^**^	1078.40^**^	325.50^**^	31.12^**^	3.95^**^	8.38^**^	2.40^*^
PUE	71.40^**^	1083.96^**^	311.00^**^	17.03^**^	4.32^**^	8.84^**^	3.81^**^
Nitrogen uptake	19.75^**^	2086.36^**^	432.58^**^	18.15^**^	6.91^**^	24.55^**^	4.53^**^
Nitrogen content	3.71^ns^	411.68^**^	16.66^**^	0.67^ns^	1.06^ns^	1.35^ns^	0.15^ns^
NUE	827.29^**^	1322.13^**^	2115.48^**^	230.82^**^	117.16^**^	207.49^**^	43.21^**^
AEN	819.49^**^	1108.27^**^	2018.08^**^	210.72^**^	132.71^**^	193.87^**^	48.49^**^

^*^ Significant at 5% probability level; ^**^ Significant at 1% probability level; ^ns^, Not significant.

**Figure 3:**
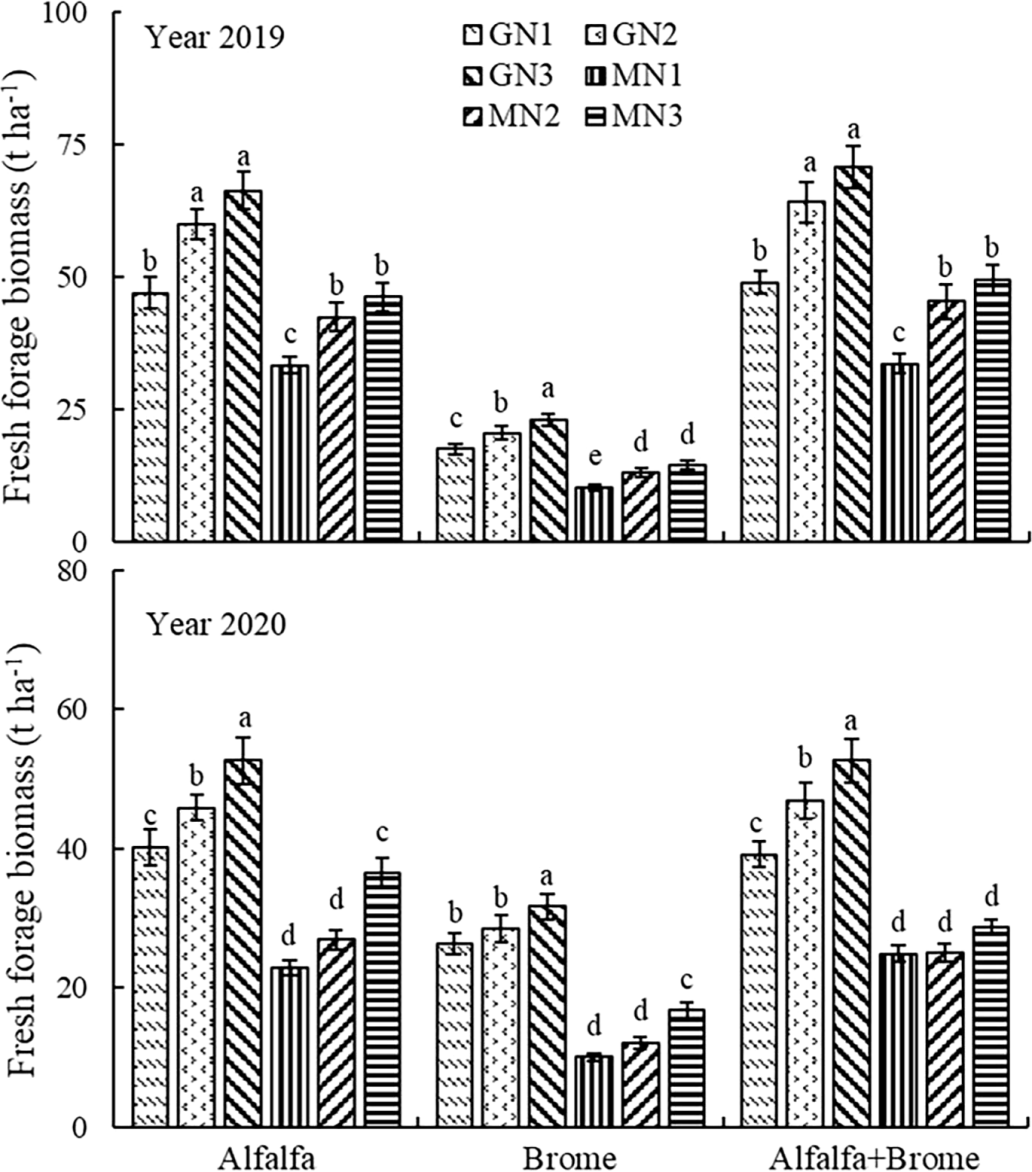
Fresh forage biomass of pasture under different treatments Note: The different lowercase letters indicate a significant difference among different treatment at *P* ≤ 0.05. GT denotes grassland type, MM denotes field management measures, and GT×MM denotes the interaction between grassland type and field management measures. The GN1, GN2, GN3, MN1, MN2, and MN3 treatments indicate no nitrogen application under grazing, 80 kg ha^-1^ nitrogen application under grazing, 160 kg ha^-1^ nitrogen applications under grazing, no nitrogen application under cutting, 80 kg ha^-1^ nitrogen application under cutting, and 160 kg ha^-1^ nitrogen application under cutting, respectively.

When analyzed the interactive effects, the GN3 treatment in alfalfa-brome mixed sowing grassland achieved the highest fresh forage biomass (70.84 and 52.60 t ha^-1^), and in alfalfa monoculture grassland (66.35 and 52.63 t ha^-1^) in 2019 and 2020, respectively (**Figure 3**). In alfalfa-brome mixed pastures, the GN3 treatment increased the fresh biomass by 44.4 and 34.3%, 10.4 and 12.3%, 110.4 and 111.7%, 56.0 and 110.2%, 42.7 and 83.7% as compared to GN1, GN2, MN1, MN2, and MN3 treatments. Whereas in alfalfa monoculture, GN3 treatment increased the fresh biomass by 41.0 and 31.0%, 10.6 and 14.8%, 98.4 and 129.8%, 56.1 and 95.8%, 43.4 and 43.9% compared to GN1, GN2, MN1, MN2, and MN3 treatments.

**Figure 4:**
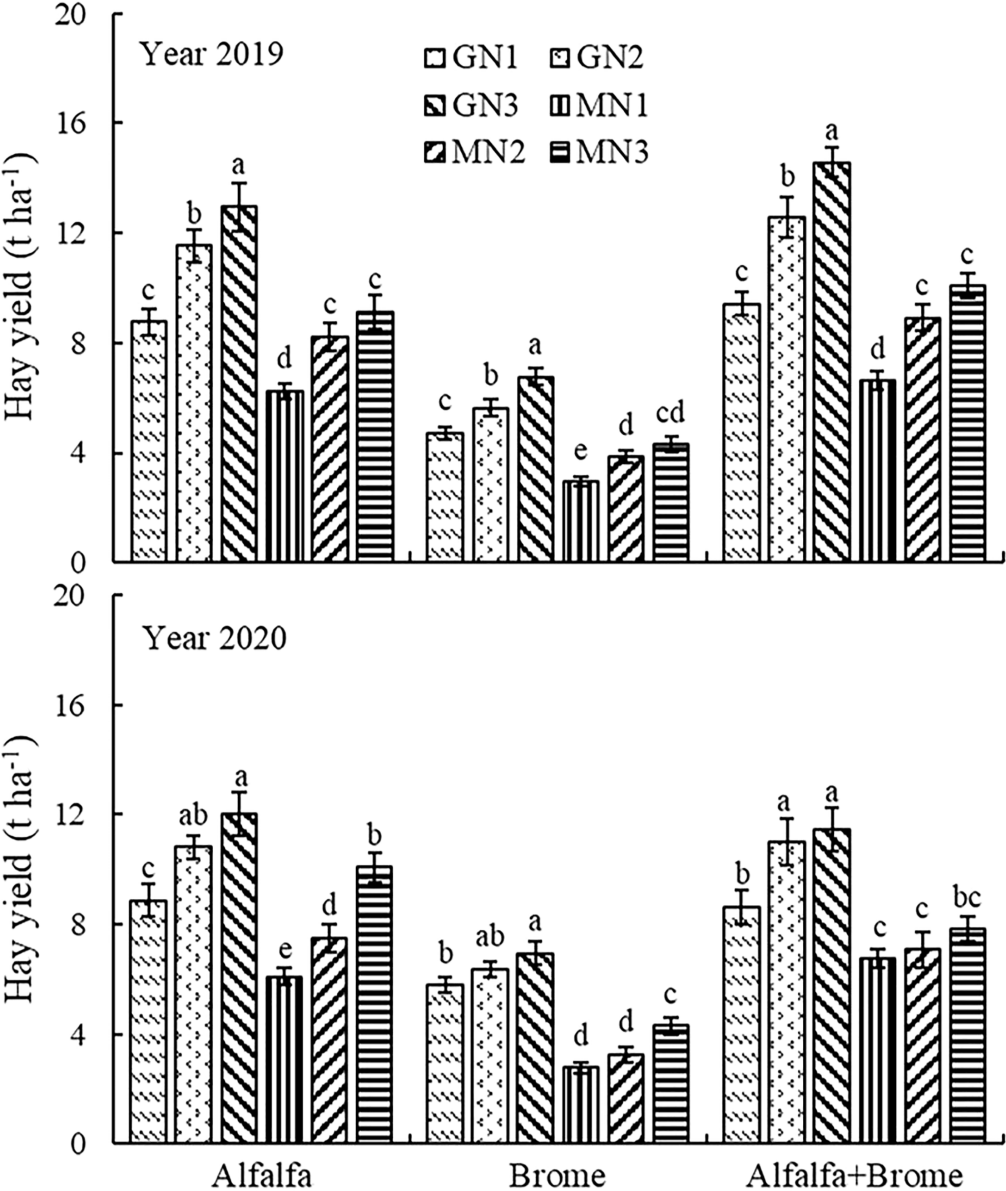
Hay yield of pasture under different treatments Note: The different lowercase letters indicate a significant difference among different treatment at *P* ≤ 0.05. GT denotes grassland type, MM denotes field management measures, and GT×MM denotes the interaction between grassland type and field management measures. The GN1, GN2, GN3, MN1, MN2, and MN3 treatments indicate no nitrogen application under grazing, 80 kg ha^-1^ nitrogen application under grazing, 160 kg ha^-1^ nitrogen application under grazing, no nitrogen application under cutting, 80 kg ha^-1^ nitrogen application under cutting, and 160 kg ha^-1^ nitrogen application under cutting, respectively.

The hay yield was significantly affected (*P* ≤ 0.01) by year (Y), grassland type (GT), and management style (MM) on of pastures (**Table 1**). The interaction effects were also significant (*P* ≤ 0.01). The hay yield of all treatments in 2019 was significantly higher by 4.5% than that in 2020. Among the grassland types, compared to brome monoculture, the hay yield in alfalfa-brome mixed sowing and alfalfa monoculture were significantly increased (**Table S1**). In addition, the GN3 showed the most significant effect among the various management styles (**Table S1**). For the interactive effects, the GN3 treatment showed the most significant effects on increasing hay yield in all grassland types compared to rest of the treatments in 2019 and 2020 (**Figure 4**). Among all the treatments, the GN3 treatment in alfalfa-brome mixed sowing grassland achieved the highest hay yield (14.58 and 11.45 t ha^-1^), and in alfalfa monoculture grassland (12.96 and 12.00 t ha^-1^) in 2019 and 2020, respectively (**Figure 4**). In alfalfa-brome mixed pastures, the GN3 treatment increased the hay yield by and 54.8%, and 32.8%, 16.0 and 4.0%, 120.2 and 69.1%, 63.5 and 61.7%, 44.8 and 46.2% as compared to GN1, GN2, MN1, MN2, and MN3 treatments. Whereas in alfalfa monoculture, GN3 treatment increased the hay yield by 47.9 and 35.4%, 12.3 and 11.0%, 107.7 and 96.7%, 57.1 and 59.8%, 41.9 and 19.2% compared to GN1, GN2, MN1, MN2, and MN3 treatments.

### 3.2 Crude protein yield

The crude protein yield was significantly affected (*P* ≤ 0.01) by year (Y), grassland type (GT), and management style (MM) on of pastures (**Table 1**). The various interaction effects were also significant (*P* ≤ 0.01). Crude protein yield of all treatments in 2019 was significantly higher by 5.4% than that in 2020. Among the grassland types, alfalfa-brome mixed sowing resulted in the highest crude protein yield whereas for management styles, GN3 showed the most significant effect (**Table S1**).

**Figure 5:**
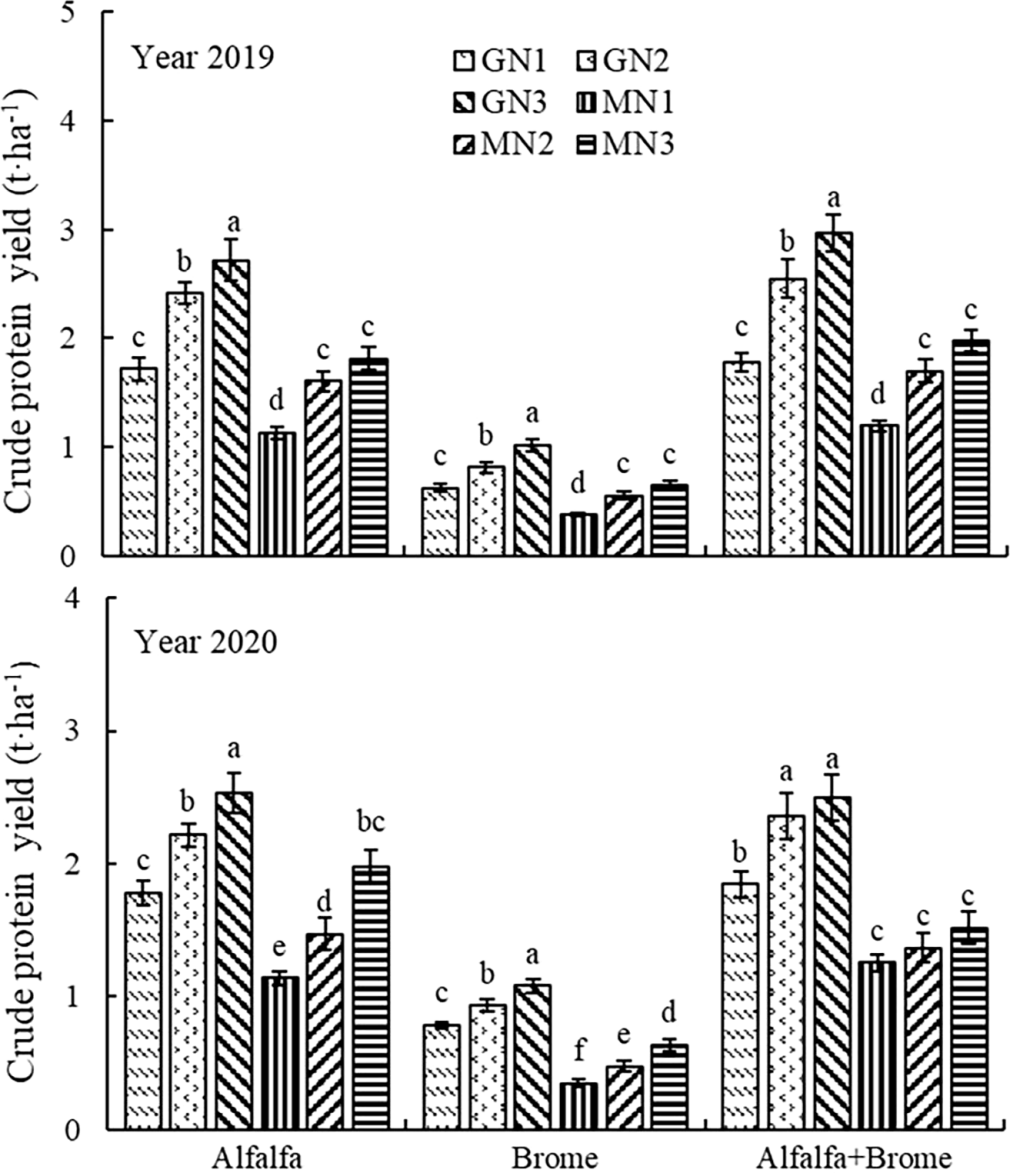
Crude protein yield of pasture under different treatments Note: The different lowercase letters indicate a significant difference among different treatment at *P* ≤ 0.05. GT denotes grassland type, MM denotes field management measures, and GT×MM denotes the interaction between grassland type and field management measures. The GN1, GN2, GN3, MN1, MN2, and MN3 treatments indicate no nitrogen application under grazing, 80 kg ha^-1^ nitrogen application under grazing, 160 kg ha^-1^ nitrogen application under grazing, no nitrogen application under cutting, 80 kg ha^-1^ nitrogen application under cutting, and 160 kg ha^-1^ nitrogen application under cutting, respectively.

When analyzed the interactive effects, the GN3 treatment showed the most significant effects on increasing crude protein yield in all grassland types, compared to other treatments in both years (**Figure 5**). Among all the treatments, the GN3 treatment in alfalfa-brome mixed sowing grassland achieved the highest crude protein yield (2.97 and 2.50 t ha^-1^), and in alfalfa monoculture grassland (2.72 and 2.53 t ha^-1^) in 2019 and 2020, respectively (**Figure 5**). In alfalfa-brome mixed pastures, the GN3 treatment increased the crude protein yield by 66.9 and 35.9%, 149.6 and 100.0%, 74.7 and 82.5%, 50.8 and 64.5% as compared to GN1, MN1, MN2, and MN3 treatments. Whereas in alfalfa monoculture, GN3 treatment increased the crude protein yield by 47.9 and 35.4%, 107.7 and 96.7%, 57.1 and 59.8%, 41.9 and 19.2% compared to GN1, MN1, MN2, and MN3 treatments.

### 3.3 Nutritional quality

Our data portrayed significant effects (*P* ≤ 0.01) of the grassland type and management style on crude protein content of forage (**Table 1**). Year (Y), GT, MM and Y×GT had a highly significant (*P* ≤ 0.01) effect on crude fat content, crude ash content, acid detergent fiber (ADF) and neutral detergent fiber (NDF) content of the forage. Y, GT and MM had highly significant (*P* ≤ 0.01) effects on RFV of forages, the interaction effects were also significant (*P* ≤ 0.01), except that of GT× MM and Y×GT×MM (**Table 1**).

The crude protein content and RFV of all treatments in 2020 was significantly higher than that in 2019. Among the grassland types, alfalfa-brome mixed sowing and alfalfa monoculture resulted in significantly higher crude protein content and RFV compared to brome monoculture (**Table S1**) In addition, the crude protein content and RFV of GN3 showed the most significant effect among the various management styles (**Table S1**). Among the interactive effects, in alfalfa monoculture and alfalfa-brome mixed sowing grassland, there was no significant difference in the crude protein content of each treatment in both years, while in brome monoculture grassland, the GN3 treatment showed significant effects on increasing crude protein content compared to GN1 treatment in both years (**Table 2**). In brome monoculture grassland, the GN3 treatment showed significant effects on increasing ether extract content compared to GN1 and MN1 treatments in both years. However, in alfalfa monoculture and alfalfa-brome mixed sowing grassland, the GN3 treatment showed significant effects on increasing ether extract content only in 2019.

**Table 2:** The content of crude protein, crude fat, crude ash, acid detergent fiber, neutral detergent fiber and relative feed value of pasture under different treatments in 2019 and 2020.

Year	Grassland type	Management style	Crude protein(%)	Crude fat(%)	Crude ash(%)	Acid detergent fiber (%)	Neutral detergent fiber (%)	Relative feed value
2019	Alfalfa	GN1	19.58a	2.84bc	13.34a	30.76ab	44.02a	139.01ab
		GN2	20.90a	3.07ab	13.90a	28.73b	40.96a	152.76a
		GN3	20.97a	3.26a	13.98a	27.73b	40.31a	157.63a
		MN1	18.15a	2.57c	13.69a	32.45a	45.06a	131.56b
		MN2	19.58a	2.91abc	14.39a	30.07ab	42.37a	143.94ab
		MN3	19.82a	3.07ab	14.53a	29.24ab	41.06a	150.67a
	Brome	GN1	13.19b	2.25bc	6.64c	32.35ab	50.18ab	118.48bc
		GN2	14.50ab	2.48ab	7.76ab	29.51bc	46.40ab	133.05ab
		GN3	15.08a	2.64a	8.08a	27.85c	44.98b	139.66a
		MN1	12.92b	2.02c	6.92bc	33.11a	51.40a	114.27c
		MN2	14.39ab	2.28bc	7.86ab	30.34abc	48.38ab	125.55abc
		MN3	15.06a	2.38ab	8.22a	29.07bc	47.15ab	130.78ab
	Alfalfa + Brome	GN1	18.91a	2.62bc	12.31a	31.01ab	45.44a	134.30bc
		GN2	20.24a	2.82ab	13.14a	28.93ab	42.11a	148.68ab
		GN3	20.36a	2.97a	13.36a	27.69b	41.32a	154.49a
		MN1	18.02a	2.43c	12.62a	32.24a	46.48a	128.66c
		MN2	19.07a	2.73abc	13.33a	29.85ab	43.28a	142.51abc
		MN3	19.58a	2.84ab	13.52a	28.72ab	42.26a	148.09ab
2020	Alfalfa	GN1	21.33ab	3.08a	11.20ab	26.73b	36.65ab	174.23bcd
		GN2	21.44ab	3.16a	10.95ab	25.64bc	35.24ab	185.64ab
		GN3	21.76a	3.71a	11.52ab	22.57c	33.99b	201.72a
		MN1	18.61b	3.07a	10.10b	30.50a	39.66a	153.18d
		MN2	19.59ab	3.08a	10.73ab	29.16ab	38.52ab	160.51cd
		MN3	19.62ab	3.66a	12.15a	26.28b	36.32ab	177.48bc
	Brome	GN1	13.54ab	2.52bc	7.23b	29.62b	53.89ab	112.71bc
		GN2	14.60a	2.64abc	7.59ab	27.46b	51.94ab	120.79ab
		GN3	15.57a	2.93a	8.33a	26.49b	48.47b	129.50a
		MN1	12.49b	2.58c	7.25b	33.67a	56.70a	100.09c
		MN2	14.58a	2.58abc	7.91ab	30.20b	52.24ab	112.23bc
		MN3	14.71a	2.74ab	8.44a	29.76b	50.25ab	121.37ab
	Alfalfa + Brome	GN1	20.11a	2.68a	11.54ab	28.22abc	37.41ab	166.19ab
		GN2	20.56a	2.73a	11.72ab	27.59bc	37.41ab	169.32ab
		GN3	21.08a	3.07a	12.19a	26.28c	35.92b	178.28a
		MN1	18.54a	2.66a	10.42b	31.68a	41.22a	144.86c
		MN2	19.30a	2.69a	10.56b	30.07ab	39.79ab	153.51bc
		MN3	19.36a	2.88a	11.21ab	29.41abc	38.61ab	159.17abc

The different lowercase letters indicate a significant difference among different treatment at *P* ≤ 0.05. The GN1, GN2, GN3, MN1, MN2 and MN3 treatments were no nitrogen applied under grazing, 80 kg ha^-1^ nitrogen applied under grazing, 160 kg ha^-1^ nitrogen applied under grazing, no nitrogen applied under cutting, 80 kg ha^-1^ nitrogen application under cutting and 160 kg ha^-1^ nitrogen application under cutting, respectively. GT denotes grassland type, MM denotes field management measures, and GT×MM denotes the interaction between grassland type and field management measures.

In brome monoculture grassland, the GN3 treatment showed significant effects on increasing crude ash content compared to GN1 and MN1 treatments in both years, while in alfalfa-brome mixed sowing grassland, the GN3 treatment showed significant effects on increasing crude ash content in 2020. In alfalfa monoculture and brome monoculture grassland, the MN1 treatment showed significant effects on increasing ADF content compared to GN2 and GN3 treatments in both years, while in alfalfa-brome mixed sowing grassland, the MN1 treatment showed significant effects on increasing ADF content compared to GN3 treatment in both years. In brome monoculture grassland, the MN1 treatment showed significant effects on increasing NDF content compared to GN3 treatment in both years, whereas in alfalfa monoculture and alfalfa-brome mixed sowing grassland, the MN1 treatment showed significant effects on increasing NDF content in 2020.

The GN3 treatment showed significant effect on increasing RFV compared to MN1 treatment in 2019 among all the grassland types, whereas in 2020, the GN3 treatment showed significant differences compared to MN1 and MN2 treatments. Among all the treatments, the GN3 treatment in alfalfa-brome mixed sowing grassland achieved the highest crude protein content (20.36 and 21.76%), and in alfalfa monoculture grassland (20.97 and 21.76%) in 2019 and 2020, respectively (**Table 2**). The GN3 treatment in alfalfa-brome mixed sowing grassland achieved the RFV (154.49 and 178.28%), and in alfalfa monoculture grassland (157.63 and 201.72%) in 2019 and 2020, respectively. In brome monoculture grassland, the GN3 treatment increased the crude protein content by 16.7 and 24.7% as compared to MN1 treatment in 2019 and 2020. In alfalfa monoculture, brome monoculture and alfalfa-brome mixed pastures, the GN3 treatment increased the RFV by 19.8, 22.2 and 20.1% as compared to MN1 treatment in 2019; GN3 treatment increased the RFV by 31.7 and 25.7%, 29.4 and 15.4%, 23.1 and 16.1% as compared to MN1 and MN2 treatments in 2020.

### 3.4 Water use status

The ANOVA results indicated significant effects (*P* ≤ 0.01) of the grassland type, management style, Y×GT and Y×MM on the soil water storage (WS) at withering and soil water storage at recovering (**Table 1**). Y and Y×GT had highly significant (*P* ≤ 0.01) effects on evapotranspiration. Y, GT and MM had highly significant (*P* ≤ 0.01) effects on water use efficiency (WUE), and precipitation use efficiency (PUE), the interaction effects were also significant (*P* ≤ 0.01). Alfalfa-brome mixed sowing and alfalfa monoculture resulted in high WUE and PUE compared to brome monoculture (**Table S1**). In addition, the WUE and PUE of GN3 showed the most significant effect among the various management styles (**Table S1**).

When analyzed the interactive effects, under three grassland types, the MN1, MN2 and MN3 treatments showed significant effects on increasing the WS at recovering and withering date compared to GN1, GN2 and GN3 treatments in alfalfa monoculture and alfalfa-brome mixed sowing grassland in 2020 (**Table 3**). In brome monoculture grassland, there was no significant difference in the WS at recovering date of all treatments, While the MN2 and MN3 treatments showed significant effects on increasing the WS at withering date compared to GN1 treatment in 2020. However, the MN1 treatment showed significant effects on increasing the WS at withering date compared to GN3 treatment in alfalfa monoculture and alfalfa-brome mixed sowing grassland in 2019. the GN3 treatment showed significant effects on increasing WUE and PUE compared to GN1, GN2, MN1, MN2 and MN3 treatments in 2019 under three grassland types. The GN2 and GN3 treatments showed significant effects on increasing WUE and PUE compared to GN1, MN1, MN2 and MN3 treatments in 2020 under three grassland types. Among all the treatments, the GN3 treatment in alfalfa-brome mixed sowing grassland achieved the highest WUE (28.49 and 27.78 kg.ha^-1.^mm^-1^), and in alfalfa monoculture grassland (24.66 and 29.84 kg.ha^-1.^mm^-1^) in 2019 and 2020, respectively (**Table 3**). the GN3 treatment in alfalfa-brome mixed sowing grassland achieved the PUE (30.48 and 28.33 kg.ha^-1.^mm^-1^), and in alfalfa monoculture grassland (27.09 and 29.70 kg.ha^-1.^mm^-1^) in 2019 and 2020, respectively. In alfalfa-brome mixed sowing grassland, the GN3 treatment increased the WUE by 52.5 and 37.6%, 114.0 and 83.6%, 61.9 and 61.4%, 42.8 and 47.5% as compared to GN1, MN1, MN2 and MN3 treatments in 2019 and 2020. Whereas the GN3 treatment increased the PUE by 54.7 and 32.8%, 120.4 and 69.2%, 63.4 and 61.7%, 44.7 and 46.2% as compared to GN1, MN1, MN2 and MN3 treatments in 2019 and 2020. In alfalfa monoculture, the GN3 treatment increased the WUE by 44.4 and 35.8%, 104.3 and 103.1%, 57.8 and 64.8%, 42.1 and 24.9% as compared to GN1, MN1, MN2 and MN3 treatments in 2019 and 2020. Whereas the GN3 treatment increased the PUE by 47.9 and 35.4%, 107.6 and 96.7%, 57.8 and 59.8%, 42.0 and 19.2% as compared to GN1, MN1, MN2 and MN3 treatments in 2019 and 2020.

**Table 3:** Soil water storage, evapotranspiration, water use efficiency (WUE) and precipitation use efficiency (PUE) under different treatments in 2019 and 2020.

Year	Grassland type	Management style	Soil water storage at recovering (mm)	Soil water storage at withering (mm)	Evapotranspiration(mm)	WUE(kg ha^-1 ^mm^-1^)	PUE(kg ha^-1 ^mm^-1^)
2019	Alfalfa	GN1	295.12a	260.27ab	513.16a	17.08c	18.32c
		GN2	287.53a	246.04ab	519.79a	22.20b	24.12b
		GN3	280.00a	232.74b	525.55a	24.66a	27.09a
		MN1	305.39a	266.40a	517.29a	12.07d	13.05d
		MN2	298.16a	251.07ab	525.39a	15.63c	17.17c
		MN3	290.59a	243.11ab	525.77a	17.36c	19.08c
	Brome	GN1	312.19a	310.93a	479.56a	9.84c	9.87c
		GN2	305.71a	296.55a	487.46a	11.57b	11.79b
		GN3	293.81a	281.36a	490.75a	13.78a	14.14a
		MN1	320.71a	316.93a	482.07a	6.08e	6.13e
		MN2	311.33a	305.92a	483.71a	7.95d	8.04d
		MN3	304.98a	289.30a	493.98a	8.72cd	9.00cd
	Alfalfa + Brome	GN1	300.52a	274.65ab	504.17a	18.68c	19.70c
		GN2	285.25a	255.26ab	508.30a	24.73b	26.28b
		GN3	273.62a	240.25b	511.68a	28.49a	30.48a
		MN1	304.01a	285.19a	497.11a	13.31d	13.83d
		MN2	296.46a	268.04ab	506.71a	17.60c	18.65c
		MN3	285.62a	259.05ab	504.87a	19.95c	21.06c
2020	Alfalfa	GN1	289.18b	241.34bc	403.36a	21.98b	21.94c
		GN2	240.60bc	216.02c	401.59a	26.91a	26.74ab
		GN3	213.51c	183.06d	402.11a	29.84a	29.70a
		MN1	181.07a	293.92a	415.34a	14.69d	15.10e
		MN2	305.16a	280.79a	414.47a	18.11c	18.58d
		MN3	291.16a	270.31ab	421.27a	23.90b	24.91bc
	Brome	GN1	287.48a	290.39b	466.78a	12.38c	14.30b
		GN2	353.07a	322.49ab	438.53ab	14.53b	15.77ab
		GN3	356.92a	336.01ab	417.37ab	16.62a	17.17a
		MN1	349.28a	331.36ab	425.30ab	6.51e	6.85d
		MN2	352.56a	364.03a	424.42ab	7.64e	8.03d
		MN3	384.35a	373.94a	402.08b	10.70d	10.64c
	Alfalfa + Brome	GN1	371.92b	225.79b	426.90a	20.19b	21.33b
		GN2	248.59b	224.04b	407.00a	27.05a	27.24a
		GN3	226.94b	214.44b	412.21a	27.78a	28.33a
		MN1	222.55a	275.39a	447.28a	15.13c	16.74c
		MN2	318.57a	290.11a	411.43a	17.21bc	17.52c
		MN3	297.44a	277.31a	415.97a	18.83b	19.38bc

The different lowercase letters indicate a significant difference among different treatment at *P* ≤ 0.05. The GN1, GN2, GN3, MN1, MN2 and MN3 treatments were no nitrogen applied under grazing, 80 kg ha^-1^ nitrogen applied under grazing, 160 kg ha^-1^ nitrogen applied under grazing, no nitrogen applied under cutting, 80 kg ha^-1^ nitrogen application under cutting and 160 kg ha^-1^ nitrogen application under cutting, respectively. GT denotes grassland type, MM denotes field management measures, and GT×MM denotes interaction between grassland type and field management measures.

### 3.5 Nitrogen utilization status

In brome monoculture grassland, the GN3 treatment showed significant effects on increasing the nitrogen content compared to MN1 treatment in 2019 (**Table 4**); while the GN3 treatment showed significant effects on increasing the nitrogen content compared to GN1 and MN1 treatments in 2020. In alfalfa monoculture grassland, the GN3 treatment showed significant effects on increasing the nitrogen content compared to MN1 treatment in 2020. In alfalfa-brome mixed sowing grassland, the GN3 treatment showed significant effects on increasing the nitrogen content compared to GN1 treatment in 2020. In alfalfa monoculture and brome monoculture grassland, the GN3 treatment showed significant effects on increasing the nitrogen uptake compared to GN1, GN2, MN1, MN2 and MN3 treatments in 2019 and 2020. In alfalfa-brome mixed sowing grassland, the GN3 treatment showed significant effects on increasing the nitrogen uptake compared to GN1, GN2, MN1, MN2 and MN3 treatments in 2019, whereas the GN3 treatment showed significant effects on increasing the nitrogen uptake compared to GN1, MN1, MN2 and MN3 treatments in 2020.

**Table 4:** Nitrogen uptake, nitrogen content, nitrogen use efficiency and agronomic efficiency of nitrogen under different treatments in 2019 and 2020.

Year	Grassland type	Management style	Nitrogen content (% )	Nitrogen uptake (kg ha^-1^)	Nitrogen use efficiency (kg kg^-1^)	Agronomic efficiency of nitrogen (kg kg^-1^)
2019	Alfalfa	GN1	3.13a	274.53c		
		GN2	3.34a	385.88b	1.39a	34.68a
		GN3	3.36a	434.81a	1.00b	26.22b
		MN1	2.90a	181.35d		
		MN2	3.13a	257.22c	0.95b	24.58b
		MN3	3.17a	289.35c	0.68c	18.01c
	Brome	GN1	2.11ab	99.55c		
		GN2	2.32ab	130.84b	0.39a	11.54a
		GN3	2.41a	163.15a	0.40a	12.78a
		MN1	2.07b	60.61d		
		MN2	2.30ab	88.53c	0.35a	11.39a
		MN3	2.41a	103.75c	0.27b	8.58b
	Alfalfa + Brome	GN1	3.03a	285.00c		
		GN2	3.24a	407.10b	1.53a	39.36a
		GN3	3.26a	474.98a	1.19b	32.24b
		MN1	2.88a	190.73d		
		MN2	3.05a	272.12c	1.02c	28.78c
		MN3	3.13a	315.53c	0.78d	21.61d
2020	Alfalfa	GN1	3.41ab	284.17c		
		GN2	3.43ab	354.93b	0.88a	24.29a
		GN3	3.48a	404.71a	0.75b	19.61b
		MN1	2.98b	181.75d		
		MN2	3.13ab	235.30d	0.67c	17.55b
		MN3	3.14ab	316.02c	0.84a	24.78a
	Brome	GN1	2.17bc	125.11c		
		GN2	2.34ab	148.89b	0.30a	7.44b
		GN3	2.49a	172.90a	0.30a	7.26b
		MN1	2.00c	55.33f		
		MN2	2.33ab	75.64e	0.25b	5.93c
		MN3	2.35ab	101.21d	0.29a	9.57a
	Alfalfa + Brome	GN1	3.22b	294.85b		
		GN2	3.29a	377.80a	1.04a	29.84a
		GN3	3.37a	399.22a	0.65b	17.68b
		MN1	2.97a	200.75d		
		MN2	3.09a	218.59cd	0.22c	3.93d
		MN3	3.10a	242.57c	0.26c	6.66c

The different lowercase letters indicate a significant difference among different treatment at *P* ≤ 0.05. The GN1, GN2, GN3, MN1, MN2 and MN3 treatments were no nitrogen applied under grazing, 80 kg ha^-1^ nitrogen applied under grazing, 160 kg ha^-1^ nitrogen applied under grazing, no nitrogen applied under cutting, 80 kg ha^-1^ nitrogen application under cutting and 160 kg ha^-1^ nitrogen application under cutting, respectively. GT denotes grassland type, MM denotes field management measures, and GT×MM denotes interaction between grassland type and field management measures.

In brome monoculture, The GN2, GN3 MN2 treatments showed significant effects on increasing the NUE and AEN compared to MN3 treatment in 2019. While in alfalfa monoculture and alfalfa-brome mixed sowing grassland, the GN2 treatment showed significant effects on increasing NUE and AEN compared to GN3 and MN2 treatments in 2019 and 2020. However, the GN2, GN3 and MN3 treatments showed significant effects on increasing NUE compared to MN2 treatment in 2020 under brome monoculture. The MN3 treatment showed significant effects on increasing AEN compared to GN2, GN3 and MN2 treatments in 2020 under brome monoculture. Among all the treatments, the GN2 treatment in alfalfa-brome mixed sowing grassland achieved the highest NUE (1.53 and 1.04 kg kg^-1^), and in alfalfa monoculture grassland (1.39 and 0.88 kg kg^-1^) in 2019 and 2020, respectively (**Table 4**). the GN3 treatment in alfalfa-brome mixed sowing grassland achieved the AEN (39.36 and 29.84 kg kg^-1^), and in alfalfa monoculture grassland (34.68 and 24.29 kg kg^-1^) in 2019 and 2020, respectively. In alfalfa-brome mixed sowing grassland, the GN2 treatment increased the NUE by 28.6 and 60%, 50 and 372.7%, 96.2 and 300% as compared to GN3, MN2, and MN3 treatments in 2019 and 2020. Whereas the GN2 treatment increased the AEN by 22.1 and 68.8%, 36.8 and 659.3%, 82.1 and 349.6% as compared to GN3, MN2 and MN3 treatments in 2019 and 2020. In alfalfa monoculture, the GN2 treatment increased the NUE by 39.0 and 17.3%, 46.3 and 31.3% as compared to GN3 and MN2 treatments in 2019 and 2020. Whereas the GN2 treatment increased the AEN by 32.3 and 23.9%, 41.1 and 38.4% as compared to GN3 and MN2 treatments in 2019 and 2020.

## 4 Discussion

### 4.1 Effects of grass-legume on forage yield, quality, water, and nitrogen utilization

Several studies have shown that the grass-legume mixed grassland results in higher forage yield than that of monoculture grassland (Nyfeler et al., 2011). Our research also shows that the fresh/hay yield of mixed cropping of grasses with alfalfa and brome was significantly higher than that of monoculture of brome. This may be due to the grass-legume mixed grassland that improved the resource utilization efficiency (such as light, moisture, and soil nutrients). Ajayi et al. (2009) reported that grass-legume mixed grassland not only increases the yield of forage but also improves the nutritional quality of forage. The results of Carpici (2017) also showed that compared with monoculture grassland, oat-pea mixed grassland have a lower content of neutral detergent fiber and acid detergent fiber, and a higher relative feed value. Our results corroborated these findings, as the two-year mixed grassland with alfalfa and brome significantly reduced the neutral detergent fiber content of the forage and increased the relative feed value, and improved the nutritional quality of forage (**Table 2**). Compared with the monoculture of gramineous or leguminous crops, grass-legume mixed grassland has advantages such as balanced feeding value, improved resource utilization efficiency, and increased forage yield (Phelan et al., 2015).

Studies have shown that mixed sowing of grass-legume increase the crude protein and dry matter yields of forages, and increases WUE to a certain extent (Zhang et al., 2018). A previous study by Dhakal et al. (2020) showed that the forage yield and nitrogen yield under alfalfa/gramineous grasses mixed sowing increased respectively by 35% and 96% compared with the monoculture of gramineous grasses. The WUE of alfalfa-gramineous mixed grassland was higher than that of monoculture of gramineous grasses, compared with the monoculture of gramineous grasses increased by 25%. Our results are similar to the findings from the above mentioned study. Under monoculture of alfalfa and mixed cropping of grasses with alfalfa and brome, the crude protein yield and WUE in both were significantly higher than those of monoculture of brome. This is due to the mixed sowing of gramineous grasses and alfalfa which can increase forage yield and increase nitrogen absorption, thereby increasing forage crude protein yield and WUE (Dhakal et al., 2020). At the same time, the strong root system of legumes forage and the shading effect of gramineous forage leaves promote the absorption and utilization of moisture by plants (Wang et al., 2015). Génard et al. (2017) showed that the nitrogen content of rape (*Brassica napus* L.) respectively intercropped with lupine (*Lupinus micranthus* Guss), clover (*Trifolium*), and Vetch (*Vicia sepium* L.) was higher than that of rape monoculture, which was increased by 34%, 140%, and 290%, respectively. The results showed that the nitrogen content of the mixed grassland of Leguminosae and Brassica was significantly higher than that of monoculture. In agreement with these finding our results also showed that the nitrogen content and NUE of the two-year alfalfa monoculture and the alfalfa- brome mixed grassland were significantly higher than those of the brome monoculture. In explanation, the biological nitrogen fixation of alfalfa not only provides a nitrogen source for its growth but also provides a nitrogen source for the growth of brome, which effectively increases the NUE of forage (Nyfeler et al., 2011).

### 4.2 Effects of grazing on forage yield, quality, and water use

Patton et al. (2007) showed that light and moderate grazing levels can increase forage yields compared to no grazing. Our research has also found that grazing significantly increased the fresh/hay yield of forages compared to cutting treatments. A possible reason for higher yield could be higher nutrient use efficiency of plants in the grazing plot compared with cutting treatments, which promotes the restoration of grassland productivity (Deléglise et al., 2015). In addition, animal manure can provide higher nutrients for grazing grassland compared with cutting management, which could be another possible reason for higher forage yields (De Boeck et al., 2010). Wilson et al. (2011) reported that pasture is frequently used under grazing management usually reduces the annual dry matter yield of pasture, but increases the crude protein content of pasture compared with cutting treatment. The results of this experiment showed that the crude protein content and yield of grazing forages were significantly higher than those of conventional cutting. This is because grazing keeps plants in the active growth and tillering stage rather than achieving the natural maturity, improving the nutritional value of forage (Bruinenberg et al., 2002).

Grazing can not only increase grassland productivity and improve forage quality but also increase the WUE of grassland (Fenetahun et al., 2020). Peng et al. (2007) found that the WUE of *Cleistogenes squarrosa*, *Agropyron cristatum*, and *Potentilla acaulis* reached the highest value under moderate grazing intensity. Our experiment results are similar to the above research, grazing significantly improves the WUE and precipitation use efficiency of forages compared with cutting treatments. This is because the trampling of livestock may increase the compaction and sealing of the soil, thereby affecting infiltration, leading to the concentration of forage roots in the surface soil, thus increasing the absorption and utilization of moisture by plant roots (Sone et al., 2020). Our research results showed that grazing significantly increased the hay yield of mixed grassland compared with cutting, but there was no significant increase in soil evapotranspiration, thus increasing the WUE of mixed grassland. The results of Zhang et al. (2020) showed that the ammonia produced by fresh chicken manure under aerobic conditions was significantly lower than that under anaerobic conditions (Schmidt et al., 2002), therefore, adding chicken grazing to rice fields significantly increased the total nitrogen content and nitrogen uptake during rice growth. The results of this experiment were similar to those of the previous study as the nitrogen content, nitrogen uptake, NUE, and AEN under the two-year grazing treatment were significantly higher than those under the conventional cutting treatment. The possible reason for these results might be the increase in soil nitrogen availability under grazing mainly through two pathways. Firstly, animal urine and feces under grazing are converted into nitrogen, so plants can more readily absorb it (Frank, 2020). Furthermore, grazing increase the labile organic compounds, stimulating microbial activity and enhancing the rate of nitrogen mineralization, and hence, inorganic nitrogen availability in the rhizosphere (Hamilton et al., 2008).

### 4.3 Effects of nitrogen application on forage yield, quality, and water use

China is among the countries with high nitrogen fertilizer usage in the world, with the average annual nitrogen fertilizer application accounting for about 30% of global nitrogen fertilizer usage (Li et al., 2003). Undeniably, nitrogen application is essential for improving the yield and quality of different crops (Varga et al., 2007; Izsaki, 2007; Kamran et al., 2022; Kamran et al., 2023). According to Ayub et al. (2009), the highest fresh yield (67.14 t ha^-1^) and hay yield (19.83 t ha^-1^) were achieved with the nitrogen application rate of 180 kg ha^-1^. Leto et al. (2008) reported that the application of 150 kg ha^-1^ nitrogen fertilizer can increase the total hay yield of forage by 9% compared with no nitrogen application. In agreement, our results indicated that 160 kg ha^-1^ nitrogen application significantly increased the fresh and hay yield of forage compared with control (no N) and 80 kg ha^-1^ nitrogen application. Szeman (2007) also found that increasing the application of nitrogen fertilizer on the grassland reduced the number and diversity of species on the grassland, but increased the forage feed value and yield of forage. However, nitrogen application shows a threshold effect in regulating crop growth and yield, i.e., excessive nitrogen application may not be conducive to the improvement of crop growth and yield (Mon et al., 2016; Kamran et al., 2023). In this experiment, the maximum nitrogen application rate (160 kg ha^-1^) may not exceed the threshold, thus as the nitrogen application rate increased, the fresh hay yield of the pasture consistently increased.

The results of Rostamza et al. (2011) also showed that nitrogen application can significantly increase the crude protein content of forages. This is because the increase in nitrogen application improves plants’ nitrogen absorption, thereby increasing the crude protein content. Our results also depicted higher crude protein content under the nitrogen treatment than that of no nitrogen application. We also found that nitrogen application significantly reduced acid and neutral detergent fiber content compared with no nitrogen application, and improved the relative feed value. This is because the increased application of nitrogen fertilizer promotes the increase of soluble substances such as protein, which accumulates in the plant cell body and leads to the dilution of the cell wall, reducing the acid detergent fiber content (Peyraud and Astigarraga, 1998). In the early autumn period, when nitrogen was applied to the grassland, the crude protein content of the forage is greater by about 20%. The combination of higher crude protein content and lower neutral detergent fiber content can further improve the nutritional quality of the forage (Méndez et al., 2019; Kamran et al., 2022). Tomić et al. (2011) found that fertilization significantly increased the crude protein yield of monoculture and mixed grassland by 194.1 and 323.2 kg ha^-1^ respectively when compared with no fertilization. Our research results are similar to Tomić et al. (2011), indicating that nitrogen application significantly increased the crude protein yield of forage compared with no nitrogen application, and the crude protein yield of forage under 160 kg ha^-1^ nitrogen application was significantly higher than that of 80 kg ha^-1^ nitrogen treatment. This may be because increased nitrogen application increased the hay yield (**Figure 4**) and crude protein content of the forage. The results of Cohen et al. (2004) also showed that the crude protein content increased with the increase of nitrogen level, which may be caused by the increase in amino acid and protein synthesis.

Several studies have reported that nitrogen application can increase the water use efficiency of cultivated grassland (Gu et al., 2017). Mariotti et al. (2015) showed that the WUE of the high-fertilizer treatment was significantly higher than that of the low-fertilizer treatment under the same grassland type. Our results are similar to the above research. The application of 160 kg ha^-1^ nitrogen significantly increased the precipitation use efficiency and WUE of forage compared with the application of 80 kg ha^-1^ nitrogen and control treatments. Because fertilization can improve water and nutrients uptake by plants, thereby maximizing the forage yield (Wang C. et al., 2018). In addition, nitrogen application improves plant growth and canopy structure, decreases direct solar radiation to the ground, reduces soil evapotranspiration (ET), and thereby increases the WUE of forage (Gu et al., 2016).

Previously, Xie et al. (2015) found that nitrogen fertilization significantly increased the nitrogen content of brome in monoculture and mixed grasslands compared with no nitrogen application. Consistently, our results showed that under the same grassland type, nitrogen application of 160 kg ha^-1^ significantly increased the nitrogen content of forage compared with no nitrogen application in both years. This may be because the nitrogen use status of forages is affected by both water and nitrogen (Soon et al., 2008). Silva et al. (2021) showed that the NUE of *Tithonia diversifolia* was the highest when nitrogen application was 100kg ha^-1^. This study showed that NUE and AEN were significantly higher with nitrogen application of 160 kg ha^-1^ than without nitrogen application and with nitrogen application of 80 kg ha^-1^. This may be due to competitive, complementary, or facilitative interactions in Grass-Legume mixed grasslands that increase the NUE of forages (Jensen et al., 2020). However, Lv et al. (2011) studied the effects of 0, 120, 240, 360, 360, 480, 600, and 720 kg ha^-1^ 7 nitrogen levels on maize and found that when the nitrogen application was higher than 360 kg ha^-1^, the agronomic efficiency, absorption and utilization rate of nitrogen fertilizer were decreased significantly. Our results differ from those reported by Lv et al. (2011). Possibly, the maximum nitrogen application (160 kg·ha^-1^) rate used in our experiment might not exceed the threshold value, and hence, the NUE of pasture showed an increase with the increase of nitrogen application rates.

## 5 Conclusion

During the two years, monoculture alfalfa and the alfalfa and brome mixed cropping resulted in insignificantly higher fresh hay yield, crude protein yield, WUE, PUE, NUE, AEN, crude protein, ether extract, and crude ash content, reduced NDF content and increase RFV compared with the monoculture of brome. The fresh hay yield, crude protein yield, WUE, PUE, NUE, and AEN of 160 kg ha^-1^ nitrogen application was significantly higher than that of no nitrogen application and 80 kg ha^-1^ nitrogen application. The application of 160 kg ha^-1^ nitrogen significantly increased the crude protein, ether extract, crude ash content, and RFV, and reduced the NDF and ADF content compared with no nitrogen application. In addition, the fresh hay yield, crude protein yield, WUE, PUE, NUE, and AEN under the grazing treatment were significantly higher than those under the conventional cutting treatment. Therefore, the mixed cropping of alfalfa and brome and nitrogen application of 160 kg ha^-1^ under grazing conditions can be adopted as an efficient management practice for improving the forage yield, nutritional quality, and water and nitrogen utilization efficiency of cultivated grassland in the Loess Plateau of China. This integrated management model is applicable to the cultivation and utilization of mixed grassland on nutrient-poor land in the Loess Plateau. However, this experiment has only been conducted for two years, Therefore, our research results and conclusions have some limitations and should be verified by long-term experiments in the future.

## Data availability statement

The raw data supporting the conclusions of this article will be made available by the authors, without undue reservation.

## Author contributions

The paper is co-authored by RX, WS, MK, SC, QJ, FH. All authors have contributed to field measurements and writing of the manuscript. All authors contributed to the article and approved the submitted version.
