# Supplementary material for: Grass-legume mixture and nitrogen application improve yield, quality, and water and nitrogen utilization efficiency of grazed pastures in the loess plateau
Source: Front Plant Sci. 2023 Jan 31;14:1088849. doi: 10.3389/fpls.2023.1088849 (PMC9940662; doi:10.3389/fpls.2023.1088849)
Supplement: Supplementary file 1 [file Table_1.pdf]

Table S1. Effect of the different treatments on forage biomass, hay yield, crude protein (CP) yield, the content of crude protein, and relative feed value (RFV); water use efficiency (WUE), precipitation use efficiency (PUE); nitrogen use efficiency (NUE) and agronomic efficiency of nitrogen (AEN) in 2019-2020

Year	Parameters	Grassland type			Management style					
		Alfalfa	Brome	Alfalfa+ Brome	GN1	GN2	GN3	MN1	MN2	MN3
2019	Forage biomass	49.27B	16.53C	52.13A	37.88C	48.24B	53.45A	25.80E	33.67D	36.81C
	Hay yield	9.47B	4.70C	10.36A	7.63C	9.92B	11.43A	5.26E	6.99D	7.84C
	CP yield	1.90B	0.67C	2.03A	1.37CD	1.92B	2.24A	0.90E	1.29D	1.48C
	CP content	19.83A	14.19B	19.36A	17.23BC	18.55A	18.80A	16.36C	17.68AB	18.15AB
	RFV	145.93A	126.97B	142.79A	130.60CD	144.83AB	150.59A	124.83D	137.34BC	143.18AB
	WUE	18.16B	9.66C	20.46A	15.20C	19.50B	22.31A	10.49E	13.73D	15.34C
	PUE	19.81B	9.83C	21.67A	15.96C	20.73B	23.90A	11.01E	14.62D	16.38C
	NUE	1.00B	0.35C	1.13A	NA	1.10A	0.86B	NA	0.77C	0.57D
	AEN	25.87B	11.07C	30.50A	NA	28.52A	23.75B	NA	21.58C	16.07D
2020	Forage biomass	37.49A	20.87B	36.19A	35.18C	40.40B	45.62A	19.25E	21.33E	27.32D
	Hay yield	9.22A	4.90C	8.79B	7.75C	9.40B	10.13A	5.21E	5.94D	7.40C
	CP yield	1.85A	0.71B	1.81A	1.47C	1.84B	2.04A	0.91E	1.10D	1.37C
	CP content	20.39A	14.25B	19.82A	18.33AB	18.87AB	19.47A	16.55C	17.82B	17.90B
	RFV	175.46A	116.11C	161.89B	151.04BC	158.58B	169.83A	132.71D	142.08C	152.67B
	WUE	22.57A	11.40C	21.03B	18.18C	22.83B	24.75A	12.11E	14.32D	17.81C
	PUE	22.83A	12.13C	21.76B	19.19C	23.25B	25.07A	12.90E	14.71D	18.31C
	NUE	0.79A	0.28C	0.54B	NA	0.74A	0.57B	NA	0.38D	0.46C
	AEN	21.56A	7.55C	14.53B	NA	20.52A	14.85B	NA	9.14D	13.67C

Note：Different uppercase letters within each factor indicate significant difference among treatment means at $P \leqslant 0.05$. the GN1, GN2, GN3, MN1, MN2 and MN3 treatments were no nitrogen applied under grazing, 80 kg ha^{-1} nitrogen applied under grazing, 160 kg ha^{-1} nitrogen applied under grazing, no nitrogen applied under cutting, 80 kg ha^{-1} nitrogen application under cutting and 160 kg ha^{-1} nitrogen application under cutting, respectively.
